# Integrated Self-Assembly of the Mms6 Magnetosome Protein to Form an Iron-Responsive Structure

**DOI:** 10.3390/ijms140714594

**Published:** 2013-07-12

**Authors:** Shuren Feng, Lijun Wang, Pierre Palo, Xunpei Liu, Surya K. Mallapragada, Marit Nilsen-Hamilton

**Affiliations:** 1 Ames National Laboratory, Ames, IA 50011, USA; E-Mail: ppalo@iastate.edu (P.P.); 2 Roy J. Carver Department of Biochemistry, Biophysics and Molecular Biology, Iowa State University, Ames, IA 50011, USA; E-Mail: sfeng@iastate.edu (S.F.); 3 Department of Chemical and Biological Engineering, Iowa State University, Ames, IA 50011, USA; E-Mails: xpliu@iastate.edu (X.L.); suryakm@iastate.edu (S.K.M.)

**Keywords:** Mms6, micelle, structural rearrangement

## Abstract

A common feature of biomineralization proteins is their self-assembly to produce a surface consistent in size with the inorganic crystals that they produce. Mms6, a small protein of 60 amino acids from *Magnetospirillum magneticum* strain AMB-1 that promotes the *in vitro* growth of superparamagnetic magnetite nanocrystals, assembles in aqueous solution to form spherical micelles that could be visualized by TEM and AFM. The results reported here are consistent with the view that the *N* and *C*-terminal domains interact with each other within one polypeptide chain and across protein units in the assembly. From studies to determine the amino acid residues important for self-assembly, we identified the unique GL repeat in the *N*-terminal domain with additional contributions from amino acids in other positions, throughout the molecule. Analysis by CD spectroscopy identified a structural change in the iron-binding C-terminal domain in the presence of Fe^3+^. A change in the intrinsic fluorescence of tryptophan in the *N*-terminal domain showed that this structural change is transmitted through the protein. Thus, self-assembly of Mms6 involves an interlaced structure of intra- and inter-molecular interactions that results in a coordinated structural change in the protein assembly with iron binding.

## 1. Introduction

Many organisms have developed the ability of creating highly ordered inorganic structures that they use for a variety of purposes ranging from structural support to magnetic guidance. Explorations of the mechanisms by which these biomineralization processes are controlled led to the identification of several biomineralization proteins with a common feature that they self-assemble into multi-molecular structures [1]. These larger structures are believed to be the functional units for biomineralization. In addition, the growth of crystals, such as occurs during biomineralization, is believed to involve movement and subsequent fusion of “islands” of minerals [2]. Being mediators of crystal growth, biomineralization proteins could be reasonably postulated to drive the movement of such mineral islands.

Mms6 is a biomineralization protein isolated from *Magnetospirillum magneticum* AMB-1 that promotes the formation of superparamagnetic magnetite particles *in vitro* [3,4] and is found associated with the magnetites of magnetosomes when isolated from these bacteria [5]. How Mms6 promotes magnetite crystal growth and the role(s) that it plays in the production of magnetosomes *in vivo* are unclear, although it has been proposed to form a scaffold in the magnetosome membrane that brings together proteins responsible for forming the magnetic particles *in vivo* [6]. In view of the frequent association with biomineralization proteins of the ability to self-assemble, it is likely that self-assembly contributes to the function of Mms6. The importance of Mms6 self-assembly is also suggested by the fact that this protein, which promotes the formation of particles of about 50 nm in diameter, is only 6000 Daltons as a monomer.

We have previously reported that Mms6 self-assembles as a micelle [7]. Mms6 is an amphiphilic protein with a hydrophobic *N*-terminal domain and a hydrophilic *C*-terminal domain. Amphiphillic proteins have been demonstrated to self-assemble into a variety of suprastrucutures, some of which are micelles [8–11]. Here we explore the nature of the self-assembly of Mms6 to better understand this protein assemblage that actively promotes the formation of magnetite nanoparticles and to determine if Mms6 is capable of movement within the larger assembly. The underlying hypothesis is that Mms6 self-assembles to form a larger structure in which individual proteins or groups of proteins are mobile with the potential of driving the fusion of mineral islands to form nanocrystals. We show that both domains contribute to stability of the micelle formed by the wild-type Mms6. By contrast, two Mms6 mutants that do not promote formation of superparamagnetic nanoparticles form less stable micelles. In addition, iron binding by the *C*-terminal domain of the wild-type, but not that of the control m3Mms6 mutant, results in a structural change that is conveyed from the *C*-terminal to the *N*-terminal domain.

## 2. Results and Discussion

### 2.1. Visualization of the Self-Assembled Mms6

**Figure 1:**
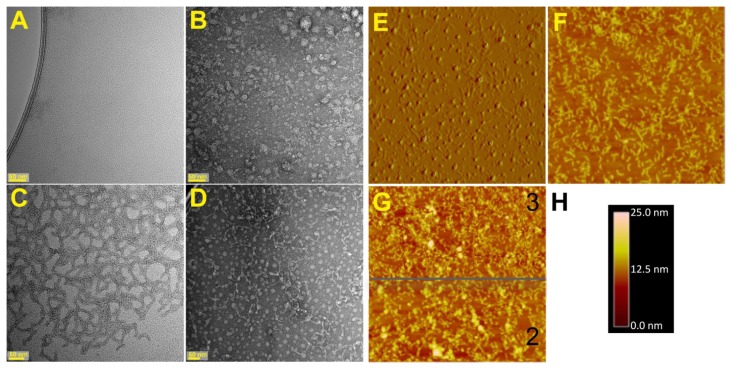
Mms6 and mutants visualized by atomic force microscopy (AFM) and transmission electron microscopy (TEM). (**A**–**D**) Negatively stained samples at 50 nm resolution of (**A**) Buffer, (**B**) Mms6, (**C**) Mms6(A131C), and (**D**) Mms6(A133C) were imaged by TEM; (**E**–**H**) AFM images of proteins immobilized on gold surfaces by a *C*-terminal cysteine; (**E**) Mms6 (A133C) amplitude image, scan area 5 μm × 5 μm, (**F**) Mms6 (A133C) height image, scan area 5 μm × 5 μm, and (**G**) Mms6(A131C) height image with two maximum scale settings (3 μm above and 2 μm below the gray line); (**H**) Scale relevant to AFM height images in (F) and (G).

Previous biochemical and biophysical analyses of Mms6, including analytical ultracentrifugation, size exclusion chromatography and protease probing suggested that Mms6 self-assembles as a micelle that can fuse with liposomes without the aid of detergents [7]. The propensity to form micelles was supported by the observation that Mms6 spreads readily on an air-water interface [12]. However, none of these methods provided information regarding the shape of the micelle and if the micelles interact. We visualized the assembled structure of Mms6 by transmission electron microscopy (TEM) using negative staining and atomic force microscopy (AFM) combined with surface immobilization (Figure 1). For this latter approach we used a mutant Mms6(A133C) (numbering of the amino acids in Mms6 is relative to the first amino acid in the ORF found in the gene encoding this protein. The mature protein is a truncated version of the translated protein due to the removal of a significant length of the *N*-terminal region of the initially translated polypeptide) in which the terminal Ala was replaced with Cys by which the protein was attached to the gold surface. Our initial premise was that the exchange of the *C*-terminal Ala for Cys would be unlikely to alter the structure or function of this protein as one strain of magnetotactic bacteria has been found to contain a sequence of Mms6 with a *C*-terminal Cys [13]. However, this expectation was not borne out as we could clearly see from the TEM images that each protein assembly is different from the others. Whereas the wild-type Mms6 appeared as spherical aggregates of a variety of sizes (Figure 1B), the Mms6-A131C appeared as a lattice of protein (Figure 1C,G) and Mms6-A133C appeared as a combination of worm-like structures and spheres (Figure 1D–F). Changing the percent hydrophobic amino acids in oleosin has been shown to alter self-assembly in anionic strength dependent manner [8]. Here the variation in protein sequence is more subtle, being a change of one hydrophobic amino acid residue for a polar residue. The possibility that the replaced cysteine might form inter-protein disulfide linkages was shown unlikely because the same images were obtained when 20 mM β-mercaptoethanol was included with each protein. The AFM images gave similar interpretations of the assembled structures of the two mutant proteins (Figure 1E–H). By TEM and dynamic light scattering (DLS) [7], we obtained two independent measures of the diameters of the wild-type protein spheres, which were 21 and 26 nm respectively. From these results we can estimate that each micelle contains 500–1000 protein molecules if they are not hollow spheres. This measured diameter is of interest with respect to the size of magnetite particle formed *in vitro* by Mms6, which is reported as ~30 nm in diameter [4].

The diameters of the Mms6(A133C) spheres were also measured by TEM and AFM as 27 and 39 nm respectively. Although apparently slightly larger, the difference between the Mms6 and Mms6(A133C) micelles was not significant as the coefficients of variation for these measurements (*N* > 80) were 14% and 18% for TEM and AFM respectively. Finally, dimensions of the Mms6(A133C) worms and the Mms6(A131C) lattice pieces were found to be the same by TEM and AFM, with the measurements being 15 × 92 nm and 16 × 86 nm respectively. Careful examination of the TEM images of Mms6 showed a number of very small particles, which had dimensions the same (14 nm) as the lattice and worm structures of the two A to C mutants. Previous DLS measurements also identified this population of smaller particles that were more numerous (94% of the particles) than the larger ones, but involved less of the total protein mass [7]. This observation brings up the possibility that these smaller Mms6 particles represent a minimal Mms6 assembly and the difference between the mutants and wild-type protein is how these structures come together to form a larger structure, which for Mms6 results in larger aggregates and for the *C*-terminal mutants results in longitudinal fusions. Regardless of the molecular relations between the observed structures, these results clearly show that the *C*-terminal domain of Mms6 is involved in determining its assembled structure.

### 2.2. Role of the *N*-Terminal Domain in Promoting Mms6 Self-Assembly

**Figure 2:**
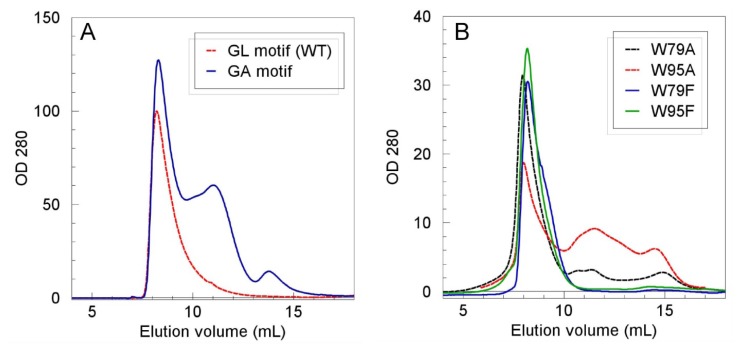
*N*-terminal domain residues important for Mms6 self-assembly. The identified Mms6 mutant proteins, all at 0.2 mg/mL, were resolved through a Superose 12 column in BC100 buffer. The molecular masses of the protein in last two peaks in panel (**A**) were estimated as 91 kDa and 36 kDa, whereas the last two peaks in panel (**B**) were estimated as 91 kDa and 20 kDa. The molecular mass of Mms6 including its His tag is 10,298 Da.

To understand the structural contributions to Mms6 self-assembly, we first examined the role of the *N*-terminal hydrophobic domain, which contains two prominent features that might contribute to the intermolecular interactions that maintain the micellar structure. These features are the two tryptophans and the GLGLGLGLGL motif that is reminiscent of the repeated motifs found in the silk proteins that mediate self-assembly [14]. However, the GL repeat is unique to a subset of magnetosome-associated proteins including Mms6, Mms7, MAM-G, MAM-D, and AMB0956. To determine if Mms6 self-assembly involves the GL repeat and the tryptophans, we prepared substitution mutants of each and tested them for their respective abilities to self-assemble. The Leu in the GL repeat was replaced with Ala to produce a protein with a GA repeat replacing the GL repeat. Each Trp was replaced separately with Ala or Phe, the latter expected to have less impact on protein structure. Self-assembly was assessed by the relative sizes of the particles as measured by size exclusion chromatography. The results showed that replacing the GL repeat with a GA repeat greatly disrupted self-assembly. Whereas, replacing either Trp with Phe, another bulky hydrophobic group, did not affect self-assembly, replacement of either Trp with Ala resulted in less stable complexes with the size distributions including smaller protein multimers and monomers (Figure 2; Table 1).

### 2.3. The *C*-Terminal Domain Contributes to the Stability of the Mms6 Micelles and Assembles in Multimeric Forms Independently of the *N*-Terminal Domain

**Figure 3:**
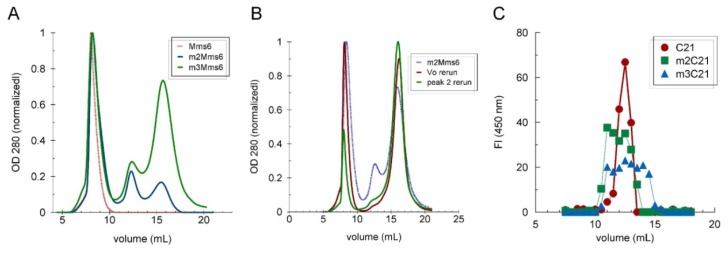
The effects of C-terminal domain mutations on Mms6 self-assembly. (**A**) Mms6 and two C-terminal domain mutants (m2- and m3Mms6), each at 1 mg/mL in BC100 buffer, were resolved by size exclusion chromatography through a Sephadex G75 column; (**B**) The void volume (first peak) and the middle peak (peak 2) of the m3Mms6 separation in (A) were separately concentrated to 0.2 mg/mL and each resolved again through the same column; (**C**) The synthetic *C*-terminal peptides of wild-type Mms6, m2- and m3Mms6 (0.2 mg/mL) were passed through a Superdex Peptide 10/300 column and quantified in each sample by o-phtalaldehyde (OPA) fluorescence. The red dashed profile for Mms6 in panel A is a repeat of a previously reported experiment [7].

For studying the ability of Mms6 to bind iron, we created two mutant proteins in which either the positions of the hydroxyl and carboxyl groups in the C-terminal domain of Mms6 were shuffled (m2Mms6) or the amino acid sequence of the C-terminal domain was scrambled (m3Mms6) [7]. Both mutants were designed to have similar hydropathy profiles as Mms6. Although all three proteins have a similar amphiphilic character only Mms6 binds iron and promotes the formation superparamagentic nanoparticles [7]. In this work, we investigated Mms6 and its m2 and m3 mutants for their abilities to self-assemble and undergo a structural change in the presence of iron and for correlations with their observed abilities to biomineralize magnetite *in vitro*. We examined the self-assembly properties of these two mutants and found that a significant percent of the protein in each case traveled as monomers and trimers compared with Mms6, which runs entirely in the void volume of the size exclusion column (Figure 3A). To determine if these forms of the protein are in equilibrium, we took individual peaks from the column and either re-ran the protein through the column (*V_o_* sample) or concentrated the protein (trimer, peak 2) and re-ran the concentrated protein through the column. The results demonstrated that these forms of the protein are in equilibrium as expected from a protein that forms a micelle with a defined CMC (Figure 3B).

Destabilization of the Mms6 assembly with C-terminal mutations suggested that the *C*-terminal domain might interact with itself. The synthetic 21 amino acid peptide *C*-terminal domain was resolved by size exclusion chromatography and found to distribute with a profile consistent with multimers. Peptides consisting of the *C*-terminal 21 amino acid sequences of the m2- or m3Mms6 distributed in less homogeneous profiles, suggesting disruption of the multimeric structure (Figure 3C). In a number of experiments and under different conditions, we determined that the C21Mms6 peptide can form multimers that range from dimers to octamers, including trimers, tetramers, heptamers and octamers. The distribution between these forms depended on the concentration of salt in the buffer and the pH and was not affected by the presence of iron (Table 2). The results clearly show that the *C*-terminal domain of Mms6 self-assembles in the absence of the *N*-terminal domain.

### 2.4. An Iron-Dependent Change in *C*-Terminal Domain Structure Is Transmitted to the *N*-terminal Domain of Mms6

**Figure 4:**
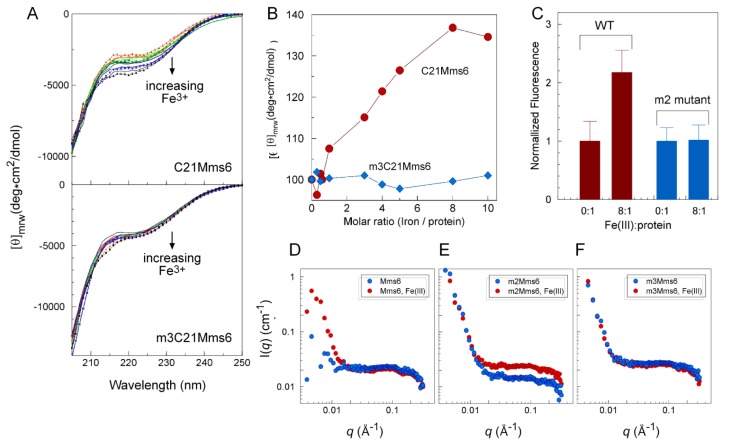
Interaction between *C*-terminal and *N*-terminal domains results in the transmission of a C-terminal domain structural change that occurs on iron binding. (**A**,**B**) CD spectra were determined for 100 μM C21Mms6 or m3C21Mms6 in 50 μM sodium formate, 100 mM KCl, pH 3.0 that had been incubated with increasing Fe^3+^:protein molar ratios for 2 h. Two independently collected data sets are included. In panel A the datasets are distinguished by one set being represented as lines and the other as markers. Due to the contribution of salts to the CD spectra below 205 nm, only the portion of the spectra above this wavelength is shown; (**C**) Intrinsic fluorescence changes measured at two molar ratios of iron:protein for Mms6 and m2Mms6, both at 5 μM; (**D**–**F**) SANS Intensity profiles with and without iron for Mms6, m2Mms6 and m3Mms6. The SANS experiment was performed only once whereas all other experiments were performed at least twice.

At pH 3, Mms6 binds iron to a high saturating stoichiometry of ~18:1 (Fe^3+^:Mms6) at saturation [7]. We examined the possibility that this binding results in a change in structure of Mms6. To determine if the *C*-terminal domain alters in conformation when it binds iron, we monitored its CD spectrum as a function of the molar ratio of iron:protein (Figure 4A,B). The results showed a change in the CD spectrum over the molar ratio of 1:1 to 8:1 (Fe^3+^:Mms6). This spectral change was not observed for the control peptide, m3C21Mms6, that does not bind iron. Thus, it appears that the *C*-terminal domain of Mms6 changes in structure upon interaction with iron. Interestingly, a similar change in CD spectrum was reported for a protein fragment from abalone shell during calcium biomineralization, which was interpreted to reflect structural re-organization of the protein upon interaction with calcium [15].

To determine if the structural change due to C-terminal domain iron binding is transmitted to the *N*-terminal domain we took advantage of intrinsic fluorescence in the *N*-terminal domain, which changed for Mms6 in the presence compared with the absence of Fe^3+^ (Figure 4C). The control mutant protein, m2Mms6, which does not bind iron, showed no change in intrinsic Trp fluorescence.

Mms6, and the two mutant proteins that do not bind iron (m2- and m3Mms6) were examined by small angle neutron scattering (SANS) for evidence of a structural change due to the binding of Fe^3+^ (Figure 4D–F). The SANS plots show differences in the low *q* region for Mms6 in the presence compared with the absence of iron, but not for m2- and m3Mms6. The SANS plots also show some differences for the m2Mms6 in the plateau area in the high *q* region, which may be due to local changes in a small length scale of m2Mms6 in the presence of iron. This study provided evidence for a shape change in the wild-type Mms6 protein assembly due to iron that was not seen for the m2- and m3Mms6 mutants. However, it should be noted that these mutations also disrupt Mms6 self-assembly, with a significant proportion of the protein appearing as monomers and trimers.

**Figure 5:**
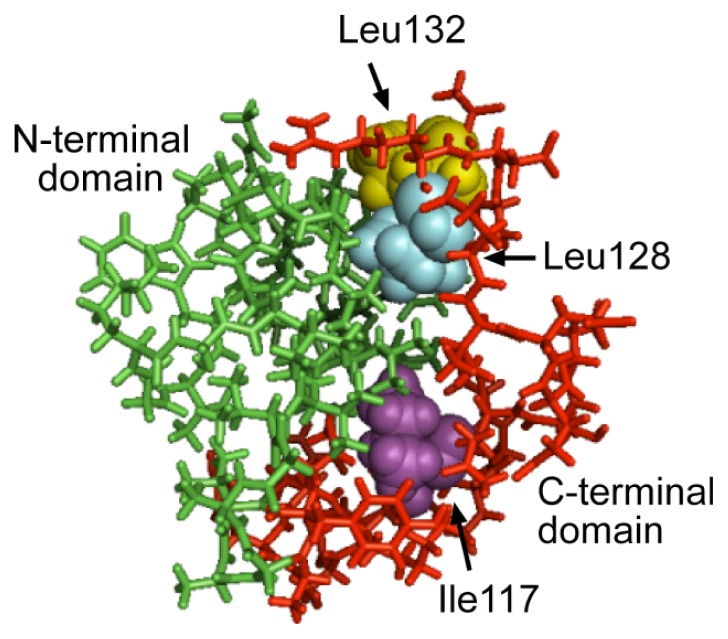
Predicted Mms6 Monomer Structure. The mature Mms6 primary sequence was entered into I-TASSER using the default parameters from the server without additional restraints. One of the predicted structures is shown as a stick model with the *N*-terminal domain as green and the *C*-terminal domain as red. This structural prediction is based on the crystal structure of chain A of d-β-hydroxybutyrate dehydrogenase (*Sinorhizobium Meliloti*; PDB 3v2Ha) which is 29% identical to Mms6 Three amino acids in the *C*-terminal domain are identified in space-filled mode with Leu128 (cyan) and Leu132 (gold) in the upper segment of the image and Ile117 (purple) in the lower portion of the image.

To identify amino acids in the C-terminal domain that might be involved in maintenance of the Mms6 assembled structure and also in mediating transmission of the structural change from *C*- to *N*-terminal domains, the Mms6 sequence was submitted to I-TASSER for a prediction of its structure (Figure 5[16,17]). From this predicted structure, it appeared that the Leu128, Leu132 and I117 in the *C*-terminal domain may interact with the *N*-terminal domain. The contributions of these amino acid residues to Mms6 self-assembly were tested by exchanging each independently with Gly and determining the effect of these mutations on the micellar integrity by size exclusion chromatography (Table 1). Whereas in many measurements under different conditions, the wild-type Mms6 remains as a large assembly that passes through the column with the void volume, mutants in which either of the two Leu was replaced with Gly resulted in a less stable structure with a significant portion of these mutant proteins were found in smaller multimers in most tests. By contrast, replacement of Ile117 with Gly did not destabilize the micelle.

## 3. Experimental Section

### 3.1. Protein Reagents and Preparation of Mutants

Expression vectors for mutant Mms6 proteins were prepared by site-directed mutagenesis of the Mms6 sequence using the Quick Change II kit from Agilent Technologies and according to the manufacturer’s instructions. Mature forms of Mms6 and its mutants as fusion proteins with histidine tags were expressed in *E. coli*, purified from inclusion bodies and refolded by dialysis [7]. Synthetic C21 peptides and peptide mutants were ordered from GenScript.

### 3.2. Atomic Force Microscopy (AFM)

Mms6(A133C) or Mms6(A131C) on a flat gold surface was scanned using AFM to explore the morphology of the protein on surface. Briefly, template-stripped gold served as the substrate, which was prepared by resistively evaporating 250 nm of gold onto a 4-in. silicon wafer with an Edwards 306A resistive evaporator. Glass microscope slides were cut into 1 × 1 cm squares and sonicated in diluted 5% Contrad 70, deionized water, and ethanol (twice), each for 30 min, and dried under a nitrogen stream. The clean glass chips were glued to the gold-coated wafer with two-part Epotek 377 (Epoxy Technology, Billerica, MA, USA) and heated at 150 °C for 1.75 h. The glass chips were then gently detached from the silicon wafer. The sandwiched gold film remained on the topside of the glass chip to yield a smooth gold surface. Three μL of 0.2 mg/mL Mms6(A133C) or Mms6(A131C) in buffer BC100 (20 mM Tris, 100 mM KCl, pH 7.5) was dropped on the gold substrate and incubated overnight at room temperature in a humid chamber created by a water-moistened filter in a sealed petri dish. The surface was then washed twice with BC100, 0.5% Tween 20 followed by two washes with 0.5% Tween 20 then dried under a nitrogen stream. AFM images were acquired using a Nanoscope III Digital Instruments/Veeco (Santa Barbara, CA, USA) in tapping mode. The diameter and length of the micelles on the reported image were obtained by measuring ~100 randomly chosen micelles.

### 3.3. Circular Dichroism (CD) Spectroscopy

Spectra were obtained with a Jasco J-710 spectropolarimeter (JASCO Corporation, Tokyo, Japan) in a 0.1 cm path-length quartz cell at 25 °C with scanning speed of 50 nm/min, resolution of 0.2 nm, bandwidth of 1.0 nm, sensitivity of 20 millidegree, time response of 8 s and average of 2 scans. Data was analyzed using JFIT (written by Bernhard Rupp, 1997, http://www.findthatzipfile.com/search-38652539-hZIP/winrar-winzip-download-cdfit.zip.htm/).

### 3.4. Fluorescence Spectroscopy

Five micromolar Mms6 or m2Mms6 in 50 mM sodium formate, 100 mM KCl, pH 3.0 were used for tryptophan fluorescence quenching measurements. Fluorescence readings (Ex: 290 nm, Em: 340 nm) were taken using a Cary Eclipse fluorescence spectrophotometer immediately after adding 40 μM FeCl_3_ (0 time) or after 2 h incubation with FeCl_3_ at 25 °C. The fluorescence values from samples of buffer under each condition with or without iron were subtracted from the values of equivalent samples containing protein. With the background thereby subtracted, these values were normalized against the “0 time” values to obtain the relative fluorescence quenching due to the interaction of each protein with Fe^3+^. The experiment was repeated 8 times and the average quenching and the standard deviation were calculated.

### 3.5. Size Exclusion Chromatography

Size exclusion chromatography was performed in an AKTA FPLC system (GE healthcare, Uppsala, Sweden) through a prepacked Superose 12 10/300GL (separation range: 1 kDa to 300 kDa), Superdex G 75 10/300GL (optimal separation range: 3 kDa to 70 kDa) and Superdex Peptide 10/300GL (optimal separation range: 7 Da to 100 kDa) columns at 4 °C. Flow rates were 0.4–0.5 mL/min. The inner dimensions of all columns were 10 × 300–310 mm (inner diameter × length) with bed volumes of 24 mL. All column samples were prepared by centrifugation at 14,000× *g* at 4 °C for 1 h. Blue dextran was used to determine the void column volume (*V*_o_) of all columns. The elution volumes (*V*_e_) of cytochrome c (MM 10.37 kDa), aprotinin (MM 6.5 kDa), insulin B chain oxidized form (MM 3495 Da) and B12 (MM 1355 Da) (all from Sigma, St. Louis, MO, USA) from a Superdex Peptide 10/300GL column were used to generate the standard curve for the apparent molecular mass estimations of C21Mms6, m2C21Mms6 and m3C21Mms6. The C21Mms6, m2C21Mms6 and m3C21Mms6 were identified using o-phtalaldehyde (OPA, Thermo Scientific, Waltham, MA, USA) by adding 200 μL of OPA to 20 μL of column fraction and measuring fluorescence (Ex: 350 nm, Em: 450 nm).

### 3.6. Small-Angle Neutron Scattering (SANS)

The SANS measurements were performed on the Low-Q Diffractometer (LQD, Lujan Center, Los Alamos National Laboratory, Los Alamos, NM, USA) of the Lujan Center at Los Alamos National Laboratory (LANL). All the solutions were made with D_2_O. One mL of 0.1 mg/mL protein was mixed with 250 μL each of 0.25 M FeCl_2_ and 0.5 M FeCl_3_ in D_2_O or with D_2_O alone. The samples were sealed in quartz banjo cells with 2 mm path lengths. The scattering vector, *q*, was varied between 0.003 and 0.3 Å^−1^, where *q* = (4π/λ)sin(θ/2) with the neutron wavelength λ and the scattering angle θ. The scattered intensity I(q) was placed on an absolute scale in the units of cm^−1^. SANS data were analyzed by software provided at the Lujan Center and corrected for empty-cell and background scattering.

### 3.7. Transmission Electron Microscopy (TEM)

Proteins were examined by transmission electron microscopy with negative staining achieved by using the single droplet procedure [18]. Briefly, 10 μL of 0.2 mg/mL protein in 2 mM Tris-HCl (pH 7.5) were individually applied to carbon coated 200 mesh copper grids. After 3 min most of the protein solution was wicked off with a filter paper and the spot covered by a droplet of fresh 2% uranyl acetate. Excess uranyl actate was removed after 30 s and the grids were air-dried at room temperature. TEM imaging was performed using a Tecnai G2 F20 Scanning Transmission Electron Microscope (STEM; FEI, Hillsboro, OR, USA) at an operating voltage of 200 kV. Multiple fields of each sample were randomly chosen and examined. Measurements of particle sizes were determined manually from electron micrograph images with at least 80 different particles measured for each determination.

## 4. Conclusions

In summary, our data is consistent with a model in which Mms6 self-assembles in micelles in a fashion that involves independent intermolecular interactions between *N*-terminal domains and between *C*-terminal domains. In addition, intramolecular interaction(s) between *N*-terminal and *C*-terminal domains are evident when the *C*-terminal domain binds iron. Thus, the Mms6 micelle can be viewed as an integrated multimolecular structure that is responsive to iron. Future studies will be directed to determine if the structural changes observed on iron binding are integral to the ability of this protein to promote the formation of magnetite crystals that are much larger in dimension than Mms6 itself but similar in size to the Mms6 protein assemblies.

## Figures and Tables

**Table 1:** The integrity of Mms6 assemblies is compromised by mutations in the *N*-terminal domain. All protein samples were loaded onto the Superose 12 column at 0.2 mg/mL protein with the exception of one sample of the GL repeat (*), which was loaded at 1 mg/mL. For each mutant protein, the distribution of protein resolved on the column and in the void volume (*V*_o_) was determined by estimating the area under the peaks using the peak integration function in the UNICORN™ software. The percent of total protein in the void volume was then calculated and is shown in the table (% protein in *V*_o_).

Mms6 protein	% protein in *V*_o_
WT	100 (15 repeats)
W79F	96, 96
W79A	79, 83
W95F	97, 97
W95A	49, 70
W79F,W95F	75, 82
L84A, L86A, L88A, L90A, L92A	46, 54, 55 *
I117G	94, 95
L128G	63, 86
L132G	29, 37

**Table 2:** Self-assembly of the *C*-terminal domain of Mms6. Eighty micrograms C21Mms6 (*C*-terminal 21 amino acids) was resolved through a Superdex Peptide 10/300 column in the presence of the buffers and other constituents as shown in the table. The sizes of the multimers, as determined from a standard curve, are reported as the average ± standard deviation with the number of independent replicates shown in parentheses.

Buffer content	# C21Mms6 units/multimer
water, pH 7.1	>75 K (2)
10 mM Pi, pH 7.5	7.3 ± 0.58 (3)
10 mM Pi, 100 mM KCl, pH 7.5	4.0 (1)
20 mM Tris, pH 7.5	2.0 ± 0.00 (3)
20 mM Tris, 100 mM KCl, pH 7.5	4.1 ± 0.14 (2)
20 mM Tris, 1.5 or 3 M KCl, pH 7.5	1.8 ± 0.38 (5)
20 mM Tris, 6 M GnHCl, pH 7.5	1.1 (1)
50 mM Formate or Citrate, pH 3	2.0 ± 0.08 (3)
50 mM Formate or Citrate, pH 3 with FeCl_3_:protein = 8:1	2.1 ± 0.15 (4)
